# Sex differences in post-operative outcomes following non-cardiac surgery

**DOI:** 10.1371/journal.pone.0293638

**Published:** 2023-11-01

**Authors:** Kai Yi Wu, Xiaoming Wang, Erik Youngson, Pishoy Gouda, Michelle M. Graham

**Affiliations:** 1 Mazankowski Alberta Heart Institute, Division of Cardiology, University of Alberta, Edmonton, Alberta, Canada; 2 Research Facilitation, Alberta Health Services, Edmonton, Alberta, Canada; Tehran University of Medical Sciences, ISLAMIC REPUBLIC OF IRAN

## Abstract

It is uncertain whether sex is an independent risk factor for poor outcomes after non-cardiac surgery. We examined sex differences in short- and long-term mortality and morbidity in patients undergoing non-cardiac surgery in Alberta, Canada. Using linked administrative databases, we identified patients undergoing one of 45 different non-cardiac surgeries who were hospitalized between 2008 and 2019. Adjusted odds ratios (95% CI) were reported for mortality at 30-days, 6-months, and 1-year stratified by sex. Secondary outcomes including all-cause hospitalization, hospitalization for heart failure (HF), hospitalization for acute coronary syndrome (ACS), hospitalization for infection, hospitalization for stroke, and hospitalization for bleeding were also analyzed. Multivariate logistic regression was adjusted for age, sex, surgery type, the components of the Charlson Comorbidity Index, and the Revised Cardiac Risk Index. We identified 552,224 unique patients who underwent non-cardiac surgery of which 304,408 (55.1%) were female. Male sex was a predictor of mortality at 30-days (aOR 1.25 (1.14, 1.38), p<0.0001), 6-months (aOR 1.26 (1.20, 1.33), p<0.0001), and 1-year (aOR 1.25 (1.20, 1.31), p<0.0001). Similarly, male sex was a predictor of hospital readmission at 30-days (1.12 (1.09, 1.14), p<0.0001), 6-months (aOR 1.11 (1.10, 1.13), p<0.0001), and 1-year (aOR 1.06 (1.04, 1.07), p<0.0001). When the results were stratified by age, the effect of male sex on clinical outcome diminished for age ≥ 65years compared to younger patients. In conclusion, male patients undergoing non-cardiac surgery have higher risks of all-cause mortality and readmission after adjustment for baseline risk factor differences, particularly in those under 65-years-old. The overall incidence of readmission for stroke, bleeding, HF and ACS after non-cardiac surgery was low. The impact of male sex on clinical outcomes decreases with increasing age, suggesting the importance of considering the effect of both sex and age on clinical outcomes after non-cardiac surgery.

## Introduction

Worldwide, approximately 100 million individuals undergo non-cardiac surgery each year [1]. In adults over the age of 45, this is an associated with a 1.8% risk of short-term mortality and a 15% risk of major complications (major bleeding, myocardial injury, or sepsis) [1]. Previous studies have described physiologic differences between males and females in relation to post operative outcomes and morbidity, thought to be due to the differential expression of sex hormones that may impact healing of acute and chronic wounds, with estrogen enhancing wound healing process [2–4]. Studies of patients undergoing non-cardiac surgery have investigated the effects of sex on perioperative outcomes such as mortality, heart failure (HF), and myocardial injury and infarction [5–9]. The results of these studies have been mixed, generally demonstrating that women have similar or lower perioperative morbidity and mortality compared to men. However, these studies were limited by small sample sizes, limited to specific surgical procedures, and often captured a non-generalizable population from an older era [5, 6].

As more patients undergo non-cardiac surgery, it is vital to understand the impact of sex on post-operative outcomes, which can lead to more patient-centric discussion of risk and inform research to improve surgical outcomes. Given the uncertainty regarding perioperative outcomes between sexes, and the lack of long-term outcome data, we aimed to examine the sex differences in perioperative mortality and morbidity in patients undergoing non-cardiac surgery in Alberta, Canada.

## Materials and methods

### Study design

A retrospective cohort of patients hospitalized for non-cardiac surgery between October 1st, 2008, and September 30th, 2020, in Alberta, Canada, was created using a series of linked administrative databases. The data was accessed on July 1st, 2022, and the authors had access to information that could identify individual patients during or after data collection. Details regarding the original cohort are published elsewhere [10, 11]. In brief, unique patient identifiers were used to link: 1) Alberta Inpatient Discharge Abstract Database, including information on all admissions to acute care facilities with most responsible admission diagnosis (coded using International Classification of Diseases, Canadian Enhancement; ICD-10) and in-patient surgical procedures (coded using Canadian Classification of Health Intervention codes, S1 and S2 Tables) The Pharmaceutical Information Network Database that captures outpatient medication dispensations in Alberta, regardless of medication insurance coverage. Over the counter medications are not captured in this database; 3) Alberta Health Services (AHS) Laboratory Database, which is the repository for all in-patient and out-patient laboratory investigations.

### Variable definitions

The age of the patient was defined as the age at the time of hospital admission. The Charlson Comorbidity Index (CCI), which includes 17 comorbidities that are associated with increased mortality, was calculated for each patient. The Revised Cardiac Risk Index (RCRI) score was calculated using administrative data, the methodology of which is previously reported [11]. Briefly, the RCRI was calculated by 1-point assignments for the presence of each of the following variables: 1) history of ischemic heart disease 2) heart failure 3) stroke or transient ischemic attack 4) insulin treated diabetes 5) creatinine ≥ 177umol/L and 6) high-risk surgery (intra-thoracic, vascular, and intra-peritoneal), for a maximum score of 6, and categorized as 0, 1, 2 (as RCRI ≤ 2), and 3 (as RCRI>2). The patient’s surgical type was separated into “Vascular”, “Intraperitoneal”, “Intrathoracic”, “Pelvic”, “Orthopedic”, “Minor” (S2 Table). The patient’s index non-cardiac surgery admission determined their surgical type. If a patient had a minor surgery and a major surgery during the same admission, they were classified as having the major surgery.

### Outcomes

The primary outcome was all-cause mortality at 30-days, 6-months, and 1-year, stratified by sex. Other outcomes of interest, for all time frames, were all-cause hospitalization, hospitalization for heart failure, hospitalization for acute coronary syndrome (ACS), hospitalization for stroke, hospitalization for bleeding, and hospitalization for infections. Subgroup analysis stratified by surgery type was also performed.

### Statistical analysis

Continuous variables were described using both mean with standard deviation (SD) and median with interval quantile range (IQR). Categorical variables were tabulated using frequency and column percentages, and p-values were from Chi-square tests. Odds ratios and 95% confidence intervals (CI) for mortality and hospital readmission were obtained using either univariate logistic regression or multivariable logistic regression that adjusted for age, RCRI (as a categorical variable), baseline Charlson comorbidities and surgical type (S1 and S2 Tables). Stepwise variable selection procedure was adopted to select important variables (with default enter and stay criterion of p <0.05). Results were also analyzed after stratifying by age ≥ 65 vs. <65 years, and by type of surgery. Statistical analysis was performed using Statistical Analysis System (SAS) Enterprise Guide 7.1 (Cary, NC, USA).

## Results

### Patient population

Between October 1, 2008, and September 30, 2020, 552,224 unique patients undergoing non-cardiac surgery were identified, of which 247,816 (44.9%) were male and 304,408 (55.1%) were female. Male patients were older (median age 56- vs. 51-years-old) and had a higher burden of preoperative comorbidities (more males with CCI category >0 (35.7% vs. 27.7%; Table 1)). Prior to surgery, males had increased history of ischemic heart disease, heart failure, atrial fibrillation, cerebrovascular disease, diabetes, hypertension, dyslipidemia, asthma, and chronic obstructive pulmonary disease (COPD) compared to female patients. There was no significant clinical difference in the median RCRI scores between sexes (median RCRI = 0 in both cohorts). The 3 most common surgeries were minor procedures (33.5%), orthopedic (25.1%), and abdominal (24.6%) surgeries. A larger proportion of female patients underwent pelvic (20.7% vs. 7.2%) than male patients, who were more likely to undergo vascular, thoracic, orthopedic, and minor surgeries (Table 1).

**Table 1:** Baseline characteristics.

	Males (N = 247,816)	Females (N = 304,408)	Overall Cohort (N = 552,224)	P value
Age				
Mean (SD)	50.2 (23.9)	50.3 (22.1)	50.3 (22.9)	< .0001
Median (IQR)	56.0 (33.0–68.0)	51.0 (36.0–67.0)	53.0 (35.0–67.0)	
RCRI Score				
Mean (SD)	0.5 (0.7)	0.4 (0.6)	0.4 (0.6)	< .0001
Median (IQR)	0.0 (0.0–1.0)	0.0 (0.0–1.0)	0.0 (0.0–1.0)	
CCI Score				
Mean (SD)	0.5 (0.9)	0.4 (0.8)	0.5 (0.8)	< .0001
Median (IQR)	0.0 (0.0–1.0)	0.0 (0.0–1.0)	0.0 (0.0–1.0)	
CCI category				
= 0	159436 (64.3%)	219897 (72.2%)	379333 (68.7%)	< .0001
= 1	55688 (22.5%)	55813 (18.3%)	111501 (20.2%)	
> = 2	32692 (13.2%)	28698 (9.4%)	61390 (11.1%)	
**Medical profile:**				
Ischemic heart disease	15261 (6.2%)	8521 (2.8%)	23782 (4.3%)	< .0001
Heart Failure	8440 (3.4%)	7489 (2.5%)	15929 (2.9%)	< .0001
Atrial Fibrillation	12728 (5.1%)	10247 (3.4%)	22975 (4.2%)	< .0001
Cerebrovascular disease	907 (0.4%)	858 (0.3%)	1765 (0.3%)	< .0001
Diabetes	33391 (13.5%)	27630 (9.1%)	61021 (11.1%)	< .0001
Chronic kidney disease	9 (0.0%)	6 (0.0%)	15 (0.0%)	0.2389
Hypertension	49284 (19.9%)	50878 (16.7%)	100162 (18.1%)	< .0001
Dyslipidemia	12518 (5.1%)	9571 (3.1%)	22089 (4.0%)	< .0001
Asthma	3091 (1.2%)	5683 (1.9%)	8774 (1.6%)	< .0001
Chronic obstructive pulmonary disease	4169 (1.7%)	2877 (0.9%)	7046 (1.3%)	< .0001
**Type of surgery**				
Vascular Surgery	4231 (1.7%)	1644 (0.5%)	5875 (1.1%)	< .0001
Abdominal Surgery	60579 (24.4%)	75306 (24.7%)	135885 (24.6%)	0.0118
Thoracic Surgery	3247 (1.3%)	2888 (0.9%)	6135 (1.1%)	< .0001
Pelvic Surgery	17830 (7.2%)	63072 (20.7%)	80902 (14.7%)	< .0001
Orthopedic Surgery	64767 (26.1%)	73815 (24.2%)	138582 (25.1%)	< .0001
Minor Surgery	97162 (39.2%)	87683 (28.8%)	184845 (33.5%)	< .0001

Note: P-values for continue/categorical variables were from Kruskal-Wallis/Chi-square test.

SD–standard deviation; IQR—interquartile range; RCRI—Revised Cardiac Risk Index; CCI—Charlson Comorbidity Index

### Outcomes

#### Mortality

The overall incidence of death at 30-days, 6-months, and 1-year was 1,828 (0.3%), 6,185 (1.1%), and 9,264 (1.7%), respectively. When stratified by sex, male patients consistently demonstrated a higher all-cause mortality compared to female patients at 30-days (0.4% versus 0.3%; p<0.0001), 6-months (1.4% versus 0.9%; p<0.0001) and 1-year (2.1% versus 1.3%; p<0.0001; Table 2).

**Table 2:** Outcomes stratified by sex.

	Outcomes	Females	Males	Total	p value
30-day	All-Cause Mortality	785 (0.3%)	1043 (0.4%)	1828 (0.3%)	< .0001
	All-Cause Hospital Readmission	18735 (6.2%)	19352 (7.8%)	38087 (6.9%)	< .0001
	Hospitalization for heart failure	1030 (0.3%)	1232 (0.5%)	2262 (0.4%)	< .0001
	Hospitalization for infection	3478 (1.1%)	3665 (1.5%)	7143 (1.3%)	< .0001
	Hospitalization for stroke	244 (0.1%)	259 (0.1%)	503 (0.1%)	0.0028
	Hospitalization for acute coronary syndrome	12 (0.0%)	18 (0.0%)	30 (0.0%)	0.0958
	Hospitalization for bleeding	19 (0.0%)	16 (0.0%)	35 (0.0%)	0.9206
6-months	All-Cause Mortality	2716 (0.9%)	3469 (1.4%)	6185 (1.1%)	< .0001
	All-Cause Hospital Readmission	44442 (14.6%)	45453 (18.3%)	89895 (16.3%)	< .0001
	Hospitalization for heart failure	2960 (1.0%)	3344 (1.3%)	6304 (1.1%)	< .0001
	Hospitalization for infection	8636 (2.8%)	9377 (3.8%)	18013 (3.3%)	< .0001
	Hospitalization for stroke	681 (0.2%)	773 (0.3%)	1454 (0.3%)	< .0001
	Hospitalization for acute coronary syndrome	42 (0.0%)	59 (0.0%)	101 (0.0%)	0.0062
	Hospitalization for bleeding	39 (0.0%)	41 (0.0%)	80 (0.0%)	0.2517
1-year	All-Cause Mortality	4101 (1.3%)	5163 (2.1%)	9264 (1.7%)	< .0001
	All-Cause Hospital Readmission	63224 (20.8%)	61282 (24.7%)	124506 (22.5%)	< .0001
	Hospitalization for heart failure	4224 (1.4%)	4729 (1.9%)	8953 (1.6%)	< .0001
	Hospitalization for infection	11781 (3.9%)	12633 (5.1%)	24414 (4.4%)	< .0001
	Hospitalization for stroke	1050 (0.3%)	1236 (0.5%)	2286 (0.4%)	< .0001
	Hospitalization for acute coronary syndrome	81 (0.0%)	105 (0.0%)	186 (0.0%)	0.0015
	Hospitalization for bleeding	59 (0.0%)	66 (0.0%)	125 (0.0%)	0.0748

**Fig 1:**
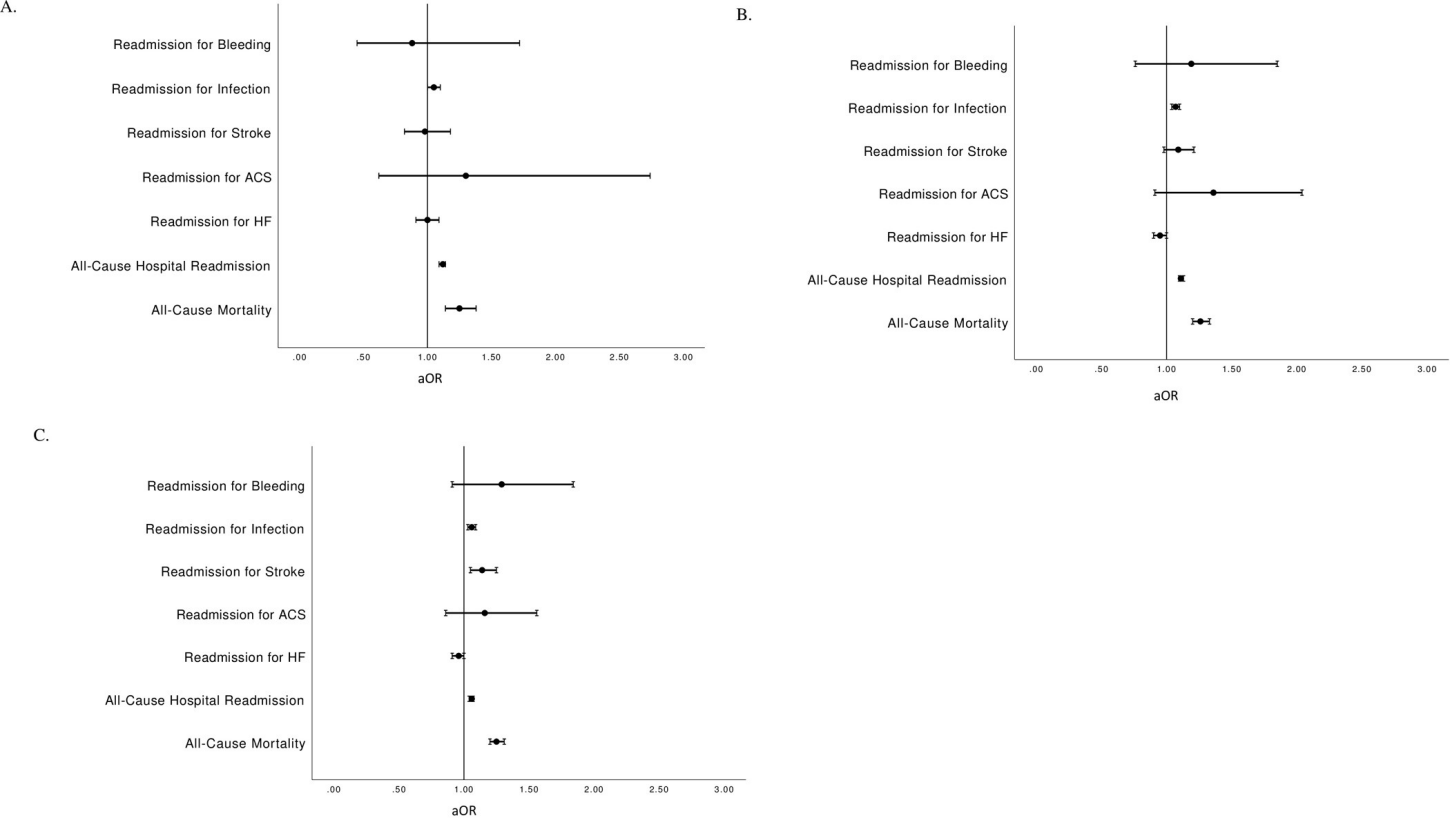
Sex-differences in clinical outcomes at 30-days (a), 6-months (b), and 1-year (c).

Following adjustment for baseline risk factor differences, male patients continued to experience a higher risk of mortality at 30-days (aOR 1.25 [1.14–1.38] p<0.0001), 6-months (aOR 1.26 [1.2–1.33], p<0.0001), and 1-year (aOR 1.25 [1.20–1.31], p<0.0001; Table 3 and Fig 1).

**Table 3:** Logistic regression (univariates) and adjusted analysis including the significant covariates from the univariate regression.

	Outcomes	Univariate		Multivariate	
		OR (95%CI)	p-value	aOR (95%CI)	p-value
30-day	All-Cause Mortality	1.64 (1.50–1.80)	< .0001	1.25 (1.14, 1.38)	< .0001
	Hospitalization for any cause	1.30 (1.27–1.33)	< .0001	1.12 (1.09, 1.14)	< .0001
	Hospitalization for heart failure	1.48 (1.36–1.61)	< .0001	1.00 (0.91, 1.09)	0.9634
	Hospitalization for infection	1.31 (1.25–1.37)	< .0001	1.05 (1.00, 1.10)	0.0652
	Hospitalization for stroke	1.31 (1.10–1.56)	0.0023	0.98 (0.82, 1.18)	0.8677
	Hospitalization for acute coronary syndrome	1.85 (0.89–3.85)	0.0977	1.30 (0.62, 2.74)	0.4821
	Hospitalization for bleeding	1.04 (0.54–2.02)	0.9062	0.88 (0.45, 1.72)	0.7072
6-months	All-Cause Mortality	1.59 (1.51–1.67)	< .0001	1.26 (1.20, 1.33)	< .0001
	Hospitalization for any cause	1.32 (1.31–1.34)	< .0001	1.11 (1.10, 1.13)	< .0001
	Hospitalization for heart failure	1.4 (1.33–1.47)	< .0001	0.95 (0.90, 1.00)	0.0739
	Hospitalization for infection	1.36 (1.32–1.4)	< .0001	1.07 (1.04, 1.10)	< .0001
	Hospitalization for stroke	1.4 (1.27–1.56)	< .0001	1.09 (0.98, 1.21)	0.1035
	Hospitalization for acute coronary syndrome	1.74 (1.17–2.58)	0.0063	1.36 (0.91, 2.04)	0.1292
	Hospitalization for bleeding	1.3 (0.84–2.01)	0.2417	1.19 (0.76, 1.85)	0.4427
1-year	All-Cause Mortality	1.57 (1.50–1.63)	< .0001	1.25 (1.20, 1.31)	< .0001
	Hospitalization for any cause	1.26 (1.25–1.28)	< .0001	1.06 (1.04, 1.07)	< .0001
	Hospitalization for heart failure	1.39 (1.33–1.45)	< .0001	0.96 (0.91, 1.00)	0.0548
	Hospitalization for infection	1.34 (1.31–1.38)	< .0001	1.06 (1.03, 1.09)	< .0001
	Hospitalization for stroke	1.46 (1.34–1.58)	< .0001	1.14 (1.05, 1.25)	0.0017
	Hospitalization for acute coronary syndrome	1.60 (1.20–2.14)	0.0014	1.16 (0.86, 1.56)	0.3285
	Hospitalization for bleeding	1.38 (0.97–1.96)	0.0706	1.29 (0.91, 1.84)	0.1525

OR- odds ratio; aOR–adjusted odds ratio

#### All-cause hospital readmission

Overall readmission was 38,087 (6.9%) at 30-days, 89,895 (16.3%) at 6-months, and 124,506 (22.5%) at 1-year (Table 2). Male patients consistently demonstrated a higher all-cause hospital readmission compared to female patients at 30-days (7.8% versus 6.2%; p<0.0001), 6-months (18.3% versus 14.16%; p<0.0001) and 1-year (24.7% versus 20.8%; p<0.0001; Table 2). This association also persisted in the adjusted analysis, with male patients demonstrating a higher risk of all-cause hospitalization at 30-days (aOR 1.12 [1.09–1.14], p<0.0001), 6-months (aOR 1.11 [0.10–1.13], p<0.0001), and 1-year (aOR 1.06 [1.04–1.07], p<0.0001, Table 3).

#### Cardiac morbidity

There were small number of ACS events at all follow-up points (30-day– 30; 6-month– 101; 1-year -186) limiting further analysis (Table 2). Although a small portion of the overall cohort was hospitalized for stroke at 1-year (0.4%), the risk of stroke at 1-year was higher in male patients (0.5%) than female patients (0.3%, aOR of 1.14 (1.05–1.25; p = 0.0017). There were insufficient stroke events for analysis at 30-days and 6-months. HF hospitalization was more frequent among male patients at all time-points (30-day: 0.5% versus 0.3%; 6-month: 1.3% versus 1.0%; 1-year: 1.9% versus 1.4%).

#### Infection

The overall incidence of readmission for infection was 7,143 (1.3%) at 30-days, 18,013 (3.3%) at 6-months, and 24,414 (4.4%) at 1-year, which accounted for 18.8%, 20.2% and 19.5% of hospitalizations, respectively. While male patients demonstrated a higher risk of 30-day hospitalization for infection in the univariate model (0.5% versus 0.3%), this did not persist in the multivariate model. However, at 6-months (aOR 1.07 [1.04–1.10], p<0.0001) and 1-year (aOR 1.06 [1.03–1.09], p<0.0001) male patients demonstrated a statistically significant difference in infection hospitalization rates (Table 3).

#### Bleeding

There were few bleeding hospitalization events at all time points (30-day– 35; 6-month– 80; 1-year -125), with no numerical difference observed between sexes (Table 2).

#### Subgroup analysis–age

We examined the clinical outcomes after stratifying by age ≥ 65 vs. <65 years (Tables 4 and S3). Male sex was a stronger predictor of each clinical outcome in those <65 than those age ≥ 65 years. Male sex was associated with reduced risk of readmission for HF and infection at 6-months in those age ≥ 65 years with aOR < 1.00.

**Table 4:** Clinical outcomes stratified by age.

	Outcomes	Age ≥ 65 years		Age < 65 years	
		aOR (95%CI)	p-value	aOR (95%CI)	p-value
30-day	All-Cause Mortality	1.19 (1.07–1.34)	0.0022	1.49 (1.24–1.78)	< .0001
	Hospitalization for any cause	1.11 (1.08–1.15)	< .0001	1.13 (1.10–1.16)	< .0001
	Hospitalization for heart failure	0.92 (0.83–1.01)	0.0804	1.23 (1.01–1.51)	0.0427
	Hospitalization for infection	0.95 (0.89–1.02)	0.1955	1.14 (1.07–1.21)	< .0001
	Hospitalization for stroke	0.99 (0.80–1.23)	0.9517	1.03 (0.74–1.43)	0.8778
	Hospitalization for acute coronary syndrome	1.28 (0.57–2.86)	0.5428	4.44 (0.52–37.8)	0.1722
	Hospitalization for bleeding	1.09 (0.35–3.36)	0.8872	1.00 (0.44–2.27)	0.9933
6-months	All-Cause Mortality	1.20 (1.12–1.28)	< .0001	1.44 (1.31–1.59)	< .0001
	Hospitalization for any cause	1.13 (1.11–1.16)	< .0001	1.09 (1.07–1.11)	< .0001
	Hospitalization for heart failure	0.91 (0.85–0.96)	0.0016	1.07 (0.95–1.21)	0.2718
	Hospitalization for infection	0.96 (0.92–1.00)	0.0747	1.16 (1.12–1.21)	< .0001
	Hospitalization for stroke	1.02 (0.90–1.15)	0.7755	1.32 (1.08–1.61)	0.0073
	Hospitalization for acute coronary syndrome	1.47 (0.92–2.34)	0.1030	1.72 (0.80–3.70)	0.1669
	Hospitalization for bleeding	1.02 (0.52–2.02)	0.9504	1.31 (0.73–2.33)	0.3669
1-year	All-Cause Mortality	1.20 (1.14–1.27)	< .0001	1.41 (1.30–1.52)	< .0001
	Hospitalization for any cause	1.12 (1.09–1.14)	< .0001	1.01 (0.99–1.03)	0.2880
	Hospitalization for heart failure	0.90 (0.86–0.95)	0.0002	1.08 (0.97–1.19)	0.1592
	Hospitalization for infection	0.95 (0.91–0.99)	0.0117	1.16 (1.12–1.20)	< .0001
	Hospitalization for stroke	1.08 (0.97–1.19)	0.1543	1.31 (1.11–1.53)	0.0012
	Hospitalization for acute coronary syndrome	1.12 (0.79–1.57)	0.5285	1.35 (0.76–2.42)	0.3107
	Hospitalization for bleeding	1.25 (0.74–2.13)	0.4043	1.30 (0.81–2.09)	0.2796

aOR–adjusted odds ratio

#### Subgroup analysis–surgery type

In patients undergoing intraperitoneal surgery, all-cause hospitalization at one-year was the only outcome that demonstrated a higher risk among females in the multivariate analysis (aOR 0.93 [0.90–0.95]; p<0.0001). Amongst vascular surgeries, males demonstrated a persistent increased risk of hospital readmission and infection in the adjusted analysis at all time points. However, no difference in all-cause mortality was observed. Only minor and pelvic surgeries consistently showed an increased risk of all-cause death and all-cause hospitalization in male patients compared to female patients at all time points. Males undergoing pelvic surgery demonstrated the greatest sex-difference, with significantly increased risk of adjusted all-cause mortality at 30-days (aOR 3.68 [2.03–6.67]; p<0.0001). Further details can be found in S4 Table, which stratifies outcomes by surgical type.

## Discussion

In this large contemporary dataset of over 500,000 patients undergoing non-cardiac surgery, we have found that male patients, compared to female patients, have a higher adjusted risk of both post-operative all-cause mortality and all-cause hospitalization at 30-days, 6-months, and 1-year. Infection was a main driver for hospitalization, with male patients having a higher adjusted risk of readmission for infections than female patients at 6-months and 1-year. Male sex was a stronger predictor of each clinical outcome in those <65 than those age ≥ 65 years.

Previous studies have evaluated sex-differences for in-hospital mortality among patients undergoing non-cardiac surgery, but very few evaluate longer term outcomes, such as mortality and readmission from 30-days to 1-year. Short-term outcomes may capture variations in outcomes that are the result of biological difference among males and females. However, ascertainment of longer-term outcomes allows for the added ability to capture variation in outcomes due to both psychosocial factors and biological differences. Previous studies with short term follow-up have identified that female sex is associated with a lower in-hospital mortality risk among patients undergoing cardiac and non-cardiac surgery [12] and that perioperative major adverse cardiovascular events occurred more among male patients [13]. Our study builds on these studies by evaluating longer-term post-operative outcomes and demonstrating a consistent increase in all-cause mortality and all-cause hospitalization among male patients.

In previous analyses of our cohort, despite adjusting for socioeconomic status and pre-operative frailty using the Hospital Frailty Risk Score (HFRS), we found that male-sex remained an independent predictor of short- and long-term mortality after non-cardiac surgery [10]. Although the present model adjusts for comorbidities using the CCI and RCRI, male sex remains an independent predictor of outcomes, which suggest that other factors could contribute to this difference. One hypothesis is that male patients tend to have fewer contacts with the primary healthcare system, resulting in surgery at later stages of disease [8]. This is supported by studies from the United States, that demonstrated that men have fewer ambulatory care visits, are less likely to have a regular family physician, and have longer interval between visits [14, 15]. This could be extrapolated to the postoperative setting, with the possibility that male patients are less likely to seek follow-up for post-operative complications.

In our study, male patients have increased rates of re-hospitalization for up to 1-year after the index surgery. In our exploratory analysis of ICD-10 codes, re-hospitalization was primarily driven by infections at all time points (S1 Fig). Although the total infection rates were low (ranging from 1.3% to 4.4% at 30-days to 1 year), male patients have increased risk of readmission for infection at 6-months and 1-year, and a trend towards increased risk at 30-days. Furthermore, infection following a surgical procedure was the most common reason for readmission at 30-days and 6-months and is the second most common reason for readmission at 1-year. Male sex has previously been shown to be a risk factor for the development of postoperative infection and those who develop a postoperative infection had higher 30-day mortality [16]. There are multiple theories for why male patients exhibit a higher incidence of infections after non-cardiac surgery [17]. Animal models demonstrate that androgens appeared to have proinflammatory effect on wounds, impairing wound-healing, whereas estrogen had an anti-inflammatory effect [3, 18]. This could also explain the change in the effect size of sex on readmission for infection in patients aged ≥ 65 years in our study. Patients in our study were admitted with surgical site as well as other infections, such as Clostridioides difficile and urinary tract infections. Therefore, the sex-differences seen are likely explained by a combination of patient factors, surgery types, and medical treatment in the perioperative period (i.e. antibiotic use).

A key finding in our study was that sex was associated with different cardiovascular outcomes, particularly readmission for heart failure, with older age. Previous studies have demonstrated that male sex and older age are independent risk factors for myocardial injury after noncardiac surgery (MINS), which is associated with major adverse cardiovascular and cerebrovascular events [13, 19, 20]. The overall incidence of readmission for ACS and heart failure after non-cardiac surgery was very low in our study. However, we demonstrated that after the age of 65-years, male sex was associated with lower readmission for heart failure in the multivariate analysis. This finding could be partially explained by hormonal changes after menopause with the loss of the protective effects of estrogen on heart disease [21, 22]. In fact, a large study of sex-age interaction on cardiometabolic risk factors in the Dutch population found that sex-differences in total cholesterol and LDL cholesterol reverse after age 55–60, when women have similar or higher lipid levels than men [23]. Our results also align with a previous study by Chu et al. which found that female sex was associated with increased perioperative HF events after intra-abdominal surgery, specifically in patients aged ≥ 65 years [7]. Further research into other possible contributors, including sex-differences in cardiac and vascular aging patterns [21] and surgical factors may help explain the sex-age interaction after non-cardiac surgery.

Non-cardiac surgery is also associated with many risk factors for stroke, such as atrial fibrillation, hypercoagulability, inflammation, sympathetic stimulation, and altered hemodynamics [24]. In the NeuroVISION study, 7% of patients aged 65 years or older who underwent inpatient elective non-cardiac surgery had covert strokes found on brain MRI done between 2 to 9 days after surgery [24]. Smilowitz et al. demonstrated that men experienced more in-hospital ischemic strokes than women after non-cardiac surgery (0.8% vs. 0.6%, aOR, 1.05 [1.04–1.07]; P < .001) [13]. We found that stroke leading to re-admission after non-cardiac surgery was infrequent in the overall cohort, with 503 (0.1%), 1454 (0.3%), and 2286 (0.4%) overall events at 30-days, 6-month, and 1-year, respectively. Male sex was associated with increased risk of readmission for stroke at 1-year after non-cardiac surgery. Although the reason for this finding is unclear, it could reflect the overall higher incidence of stroke among males in general rather than association with surgery itself [25].

### Limitations

This study does have limitations. First, our study relies on ICD coding for outcomes, which has inherent issues with bias or coding errors. However, our large sample size of patients across the entire province of Alberta may help mitigate some of these limitations. Secondly, using large sample sizes, smaller effect sizes may be statically significant. Therefore, the clinical relevance of small effect sizes should be considered when interpretating these significant results. Thirdly, our sample includes all patients who were hospitalized for non-cardiac surgery rather than just limited to elective surgeries. Fourthly, minor surgery, orthopedic surgery and abdominal surgery constitute most of the surgeries in our study. Therefore, the results may be more applicable to these surgeries than other procedures, especially minor procedures. Sex differences in mortality and re-hospitalization depend on the type of procedure and are likely related to multiple surgical, anesthetic, and medical factors. This analysis is hypothesis generating but future research into sex differences for each class of surgery is required.

## Conclusion

In patients who undergo non-cardiac surgery, male sex is an independent predictor of all-cause mortality and re-hospitalization at 30-days, 6-month, 1-year, which is driven by increased infections related to the procedure. The overall short- and long-term incidence of readmission for stroke, bleeding, heart failure and ACS after non-cardiac surgery was low. The impact of male sex on clinical outcomes decreases with increasing age, which suggests the importance of considering both sex and age on clinical outcomes after non-cardiac surgery. Further work to explore both the biological and psychosocial factors behind this observed increased risk is required.

## Supporting information

S1 Table Included surgical procedures. (PDF)

S2 Table Surgery type classification. (PDF)

S3 Table Outcomes stratified by age. (PDF)

S4 Table Outcomes stratified by the type of surgery. (PDF)

S1 Fig Causes of re-hospitalization at 30-days, 6-month, and 1-year based on ICD-10 codes. (PDF)
