# Supplementary material for: Sex differences in post-operative outcomes following non-cardiac surgery
Source: PLoS One. 2023 Nov 1;18(11):e0293638. doi: 10.1371/journal.pone.0293638 (PMC10619824; doi:10.1371/journal.pone.0293638)
Supplement: S1 Table — (PDF) [file pone.0293638.s001.pdf]

S1 Table – Included Surgical Procedures

GI endoscopic	1NA56BA, 1NE50BA, 1NF13BA, 1NM50BA, 1NM56BA, 1NQ56BA, 2NA70BA, 2NA71BP, 2NA71BR, 2NC70BA, 2NF70BA, 2NF71BA, 2NK70BA, 2NK71BA, 2NK71BR, 2NM70BA, 2NM71BA, 2NM71BR, 2NQ70BA, 2NQ71BA
Bronchoscopy	1GJ56BA, 1GM56BA, 1GT56DA, 2GM70BA, 2GM71BA, 2GM71BR, 2GT71BA, 2GT71BP
Fixation of fracture, radius and ulna	1TV74
Fixation of fracture, tibia and fibular	1VQ74
Cystoscopy, TURP, TURBT	1PE56BA, 1PL72BA, 1PM56BA, 1PM87BA, 1PQ50BA, 1PQ56BA, 1QT87BA, 2PG70DA, 2PG71BA, 2PG71DA, 2PG71BR, 2PM70BA, 2PM71BA, 2PM71DA, 2PQ70BA, 2PQ71BA, 2QT70BA, 2QT71BA
Cataract	1CL89VRLM
Abdominal wall hernia repair	1SY80
Mastectomy and superficial procedures	1YA, 1YB, 1YC, 1YD, 1YE, 1YF, 1YG, 1YH, 1YI, 1YJ, 1JK, 1YL, 1YM, 1YN, 1YO, 1YP, 1YQ, 1YR, 1YS, 1YT, 1YU, 1YV, 1YW, 1YX, 1YY, 1YZ
Appendectomy	1NV89
Oophorectomy	1RB57, 1RB87, 1RB89
Salpingo-oopherectomy	1RD89
Splenectomy	1OB87, 1OB89
Gastrectomy	1NF87DG, 1NF82RJ, 1NF87DH, 1NF87DJ, 1NF87DL, 1NF87DQ, 1NF87LA, 1NF87RG, 1NF87RH, 1NF87RJ, 1NF87RK, 1NF87RP, 1NF87SH
Hysterectomy	1RM87, 1RM89, 1RM 89
Resection of small intestine	1NK87DA, 1NK87DN, 1NK87DP, 1NK87DX, 1NK87DY, 1NK87LA, 1NK87RE, 1NK87RF, 1NK87TF, 1NK87TG
Below-knee amputation	1VQ93LA
Above-knee amputation	1VC93LA
Metatarsal amputation	1WJ93LA
ORIF femur	1VC74
Knee arthroplasty	1VG53, 1VG80
Hip arthroplasty	1VA53, 1VA74

Ankle ORIF	1WA74LANW, 1WA73LA, 1WA80
Rotator cuff repair	1TC80, 1TV80
Total gastrectomy	1NF89DZ, 1NF89GW, 1NF89SG, 1NF89TH, 1NF91RG, 1NF91RJ, 1NF91RP, 1NF91SG
Cholecystectomy	1OD52, OD57, 1OD80, 1OD89
AV fistula repair	1KY76LA
Nephrectomy	1PC87, 1PC89, 1PC91, 1PM87, 1PM89, 1PM91
Hysterectomy	1RM89, 1RM91
Carotid endarterectomy	1JE57LA
Aortofemoral bypass	1KA76MZ
Femoral-popliteal bypass	1KG76MI
Partial colectomy	1NM87
Total colectomy	1NM89, 1NM91
Bowel obstruction	1NP72
Lysis of abdominal adhesions	1OT72
Lobectomy	1GR87, 1GR89, 1GR91, 1GT87
Pneumonectomy	1GR89, 1GT89, 1GT91
Discectomy	1SE89PF
Spinal fusion	1SC75PFNWA
Cruciate ligament repair	1VL80
AAA repair	1KA80LA
Pancreatectomy	1OJ87, 1OK89
Cystectomy	1PM57,1PM80, 1PM84, 1PM87, 1PM89, 1PM90, 1PM91, 1PM92
Prostatectomy	1QT91
Spinal vertebral repair	1SC80PF
