# Supplementary material for: Sex differences in post-operative outcomes following non-cardiac surgery
Source: PLoS One. 2023 Nov 1;18(11):e0293638. doi: 10.1371/journal.pone.0293638 (PMC10619824; doi:10.1371/journal.pone.0293638)
Supplement: S2 Table — (PDF) [file pone.0293638.s002.pdf]

S2 Table – Surgery type classification

Vascular	Carotid endarterectomy, AAA repair, Aorto-femoral bypass, Femoral-popliteal bypass, AV fistula repair
Intraperitoneal	Partial gastrectomy, Total gastrectomy, Resection of small intestine, Partial colectomy, Total colectomy, Bowel obstruction, Appendectomy, Splenectomy, Pancreatectomy, Nephrectomy
Intrathoracic	Lobectomy, Pneumonectomy
Pelvic	Nephrectomy, Prostatectomy, Oopherectomy, Salpingo-oopherectomy, Hysterectomy, Cystectomy
Orthopedic	Spinal vertebral repair, Discectomy, Spinal fusion, Below-knee amputation, Above-knee amputation, Metatarsal amputation, ORIF femur, Knee arthroplasty, Hip arthroplasty, Ankle ORIF
Minor	GI endoscopic procedures, Cystoscopy, TURP, TURBT, Cataract, Mastectomy and superficial procedures, endoscopic respiratory procedures
