# Supplementary material for: Sex differences in post-operative outcomes following non-cardiac surgery
Source: PLoS One. 2023 Nov 1;18(11):e0293638. doi: 10.1371/journal.pone.0293638 (PMC10619824; doi:10.1371/journal.pone.0293638)
Supplement: S3 Table — (PDF) [file pone.0293638.s003.pdf]

S3 Table: Outcomes Stratified by Age

	Outcomes	<65 years	≥65 years	Total	p value
30-day	All-Cause Mortality	551 (0.1%)	1277 (0.8%)	1828 (0.3%)	<.0001
	All-Cause Hospital Readmission	22880 (5.9%)	15207 (9.2%)	38087 (6.9%)	<.0001
	Hospitalization for heart failure	450 (0.1%)	1812 (1.1%)	2262 (0.4%)	<.0001
	Hospitalization for infection	4014 (1.0%)	3129 (1.9%)	7143 (1.3%)	<.0001
	Hospitalization for stroke	152 (0.0%)	351 (0.2%)	503 (0.1%)	<.0001
	Hospitalization for ACS	6 (0.0%)	24 (0.0%)	30 (0.0%)	<.0001
	Hospitalization for bleeding	23 (0.0%)	12 (0.0%)	35 (0.0%)	0.5741
6-months	All-Cause Mortality	2048 (0.5%)	4137 (2.5%)	6185 (1.1%)	<.0001
	All-Cause Hospital Readmission	51861 (13.4%)	38034 (23.0%)	89895 (16.3%)	<.0001
	Hospitalization for heart failure	1277 (0.3%)	5027 (3.0%)	6304 (1.1%)	<.0001
	Hospitalization for infection	9790 (2.5%)	8223 (5.0%)	18013 (3.3%)	<.0001
	Hospitalization for stroke	415 (0.1%)	1039 (0.6%)	1454 (0.3%)	<.0001
	Hospitalization for ACS	28 (0.0%)	73 (0.0%)	101 (0.0%)	<.0001
	Hospitalization for bleeding	47 (0.0%)	33 (0.0%)	80 (0.0%)	0.0271
1-year	All-Cause Mortality	3090 (0.8%)	6174 (3.7%)	9264 (1.7%)	<.0001
	All-Cause Hospital Readmission	71668 (18.5%)	52838 (32.0%)	124506 (22.5%)	<.0001
	Hospitalization for heart failure	1854 (0.5%)	7099 (4.3%)	8953 (1.6%)	<.0001
	Hospitalization for infection	12977 (3.4%)	11437 (6.9%)	24414 (4.4%)	<.0001
	Hospitalization for stroke	649 (0.2%)	1637 (1.0%)	2286 (0.4%)	<.0001
	Hospitalization for ACS	49 (0.0%)	137 (0.1%)	186 (0.0%)	<.0001
	Hospitalization for bleeding	70 (0.0%)	55 (0.0%)	125 (0.0%)	0.0006
