# Supplementary material for: Sex differences in post-operative outcomes following non-cardiac surgery
Source: PLoS One. 2023 Nov 1;18(11):e0293638. doi: 10.1371/journal.pone.0293638 (PMC10619824; doi:10.1371/journal.pone.0293638)
Supplement: S4 Table — (PDF) [file pone.0293638.s004.pdf]

	Outcome (Vascular Surgery)	Event rate (%)		Univariate		Multivariate	
		**Male**	**Female**	**OR (95% CI)**	**p-value**	**OR (95% CI)**	**p-value**
**30-day**	All-Cause Mortality	11 (0.3%)	5 (0.3%)	0.85 (0.30,2.46)	0.7709	0.85 (0.30,2.46)	0.7709
	All-Cause Hospital Readmission	350 (8.3%)	186 (11.3%)	0.71 (0.59,0.85)	0.0003	0.69 (0.57,0.83)	<.0001
	Hospitalization for heart failure	23 (0.5%)	14 (0.9%)	0.64 (0.33,1.24)	0.1840	0.66 (0.34,1.29)	0.2252
	Hospitalization for infection	58 (1.4%)	49 (3.0%)	0.45 (0.31,0.66)	<.0001	0.42 (0.29,0.62)	<.0001
	Hospitalization for stroke	27 (0.6%)	12 (0.7%)	0.87 (0.44,1.73)	0.6976	1.00 (0.50,1.98)	0.9914
	Hospitalization for ACS	0 (0.0%)	0 (0.0%)				
	Hospitalization for bleeding	0 (0.0%)	0 (0.0%)				
**6-months**	All-Cause Mortality	36 (0.9%)	20 (1.2%)	0.70 (0.40,1.21)	0.1977	0.70 (0.40,1.21)	0.1977
	All-Cause Hospital Readmission	918 (21.7%)	408 (24.8%)	0.84 (0.73,0.96)	0.0103	0.81 (0.71,0.93)	0.0023
	Hospitalization for heart failure	64 (1.5%)	35 (2.1%)	0.71 (0.47,1.07)	0.1011	0.71 (0.47,1.09)	0.1208
	Hospitalization for infection	152 (3.6%)	101 (6.1%)	0.57 (0.44,0.74)	<.0001	0.53 (0.41,0.69)	<.0001
	Hospitalization for stroke	48 (1.1%)	20 (1.2%)	0.93 (0.55,1.57)	0.7918	1.04 (0.61,1.76)	0.8872
	Hospitalization for ACS	1 (0.0%)	0 (0.0%)				
	Hospitalization for bleeding	0 (0.0%)	0 (0.0%)				
**1-year**	All-Cause Mortality	62 (1.5%)	30 (1.8%)	0.80 (0.52,1.24)	0.3201	0.78 (0.50,1.21)	0.2587
	All-Cause Hospital Readmission	1258 (29.7%)	556 (33.8%)	0.83 (0.73,0.94)	0.0024	0.80 (0.71,0.91)	0.0004
	Hospitalization for heart failure	103 (2.4%)	54 (3.3%)	0.73 (0.53,1.03)	0.0707	0.76 (0.54,1.07)	0.1196
	Hospitalization for infection	206 (4.9%)	139 (8.5%)	0.55 (0.44,0.69)	<.0001	0.52 (0.41,0.65)	<.0001
	Hospitalization for stroke	67 (1.6%)	30 (1.8%)	0.87 (0.56,1.34)	0.5151	0.94 (0.61,1.46)	0.7911
	Hospitalization for ACS	2 (0.0%)	0 (0.0%)				
	Hospitalization for bleeding	1 (0.0%)	1 (0.1%)				

aOR – adjusted odds ratio

Vascular

	Outcome (Intraperitoneal Surgery)	Event rate (%)		Univariate		Multivariate	
		Male	Female	OR (95% CI)	p-value	OR (95% CI)	p-value
**30-day**	All-Cause Mortality	111 (0.2%)	110 (0.1%)	1.25 (0.96,1.63)	0.0918	1.03 (0.79,1.35)	0.8048
	All-Cause Hospital Readmission	4150 (6.9%)	4732 (6.3%)	1.10 (1.05,1.15)	<.0001	1.02 (0.98,1.07)	0.3448
	Hospitalization for heart failure	167 (0.3%)	161 (0.2%)	1.29 (1.04,1.60)	0.0212	0.89 (0.71,1.12)	0.3219
	Hospitalization for infection	681 (1.1%)	719 (1.0%)	1.18 (1.06,1.31)	0.0021	1.03 (0.93,1.15)	0.5783
	Hospitalization for stroke	35 (0.1%)	40 (0.1%)	1.09 (0.69,1.71)	0.7163	0.92 (0.58,1.46)	0.7279
	Hospitalization for ACS	5 (0.0%)	4 (0.0%)	1.55 (0.42,5.79)	0.5111	1.25 (0.33,4.73)	0.7431
	Hospitalization for bleeding	3 (0.0%)	3 (0.0%)	1.24 (0.25,6.16)	0.7899	1.24 (0.25,6.16)	0.7899
**6-months**	All-Cause Mortality	452 (0.7%)	407 (0.5%)	1.38 (1.21,1.58)	<.0001	1.14 (0.99,1.31)	0.0655
	All-Cause Hospital Readmission	8513 (14.1%)	9706 (12.9%)	1.11 (1.07,1.14)	<.0001	1.00 (0.97,1.04)	0.8619
	Hospitalization for heart failure	464 (0.8%)	411 (0.5%)	1.41 (1.23,1.61)	<.0001	0.97 (0.84,1.11)	0.6315
	Hospitalization for infection	1551 (2.6%)	1579 (2.1%)	1.23 (1.14,1.32)	<.0001	1.05 (0.98,1.13)	0.1554
	Hospitalization for stroke	131 (0.2%)	99 (0.1%)	1.65 (1.27,2.14)	0.0002	1.30 (1.00,1.69)	0.0544
	Hospitalization for ACS	10 (0.0%)	6 (0.0%)	2.07 (0.75,5.70)	0.1586	1.53 (0.55,4.26)	0.4190
	Hospitalization for bleeding	6 (0.0%)	7 (0.0%)	1.07 (0.36,3.17)	0.9093	1.07 (0.36,3.17)	0.9093
**1-year**	All-Cause Mortality	714 (1.2%)	664 (0.9%)	1.34 (1.21,1.49)	<.0001	1.12 (1.00,1.25)	0.0423
	All-Cause Hospital Readmission	11277 (18.6%)	13739 (18.2%)	1.02 (1.00,1.05)	0.0793	0.93 (0.90,0.95)	<.0001
	Hospitalization for heart failure	639 (1.1%)	586 (0.8%)	1.36 (1.21,1.52)	<.0001	0.96 (0.85,1.08)	0.4745
	Hospitalization for infection	2081 (3.4%)	2203 (2.9%)	1.18 (1.11,1.25)	<.0001	1.01 (0.95,1.08)	0.7061
	Hospitalization for stroke	212 (0.3%)	146 (0.2%)	1.81 (1.46,2.23)	<.0001	1.45 (1.17,1.80)	0.0007
	Hospitalization for ACS	16 (0.0%)	8 (0.0%)	2.49 (1.06,5.81)	0.0354	1.79 (0.76,4.240)	0.1835
	Hospitalization for bleeding	10 (0.0%)	13 (0.0%)	0.96 (0.42,2.18)	0.9153	0.96 (0.42,2.18)	0.9153

aOR – adjusted odds ratio

Intraperitoneal

	Outcome (Intrathoracic Surgery)	Event rate (%)		Univariate		Multivariate	
		**Male**	**Female**	**OR (95% CI)**	**p-value**	**OR (95% CI)**	**p-value**
**30-day**	All-Cause Mortality	9 (0.3%)	4 (0.1%)	2.00 (0.62,6.51)	0.2478	1.84 (0.56,6.08)	0.3167
	All-Cause Hospital Readmission	221 (6.8%)	186 (6.4%)	1.06 (0.87,1.30)	0.5655	1.14 (0.93,1.40)	0.2085
	Hospitalization for heart failure	5 (0.2%)	6 (0.2%)	0.74 (0.23,2.43)	0.6206	0.67 (0.20,2.23)	0.5140
	Hospitalization for infection	31 (1.0%)	25 (0.9%)	1.10 (0.65,1.87)	0.7143	1.08 (0.63,1.84)	0.7796
	Hospitalization for stroke	3 (0.1%)	2 (0.1%)	1.33 (0.22,7.99)	0.7522	1.33 (0.22,7.99)	0.7522
	Hospitalization for ACS	0 (0.0%)	0 (0.0%)				
	Hospitalization for bleeding	1 (0.0%)	0 (0.0%)				
**6-months**	All-Cause Mortality	58 (1.8%)	29 (1.0%)	1.79 (1.14,2.81)	0.0107	1.68 (1.07,2.65)	0.0250
	All-Cause Hospital Readmission	617 (19.0%)	520 (18.0%)	1.07 (0.94,1.22)	0.3160	1.17 (1.03,1.34)	0.0201
	Hospitalization for heart failure	16 (0.5%)	16 (0.6%)	0.89 (0.44,1.78)	0.7397	0.77 (0.38,1.56)	0.4743
	Hospitalization for infection	88 (2.7%)	91 (3.2%)	0.86 (0.64,1.15)	0.3063	0.86 (0.64,1.15)	0.3063
	Hospitalization for stroke	12 (0.4%)	7 (0.2%)	1.53 (0.60,3.88)	0.3744	1.53 (0.60,3.88)	0.3744
	Hospitalization for ACS	0 (0.0%)	0 (0.0%)				
	Hospitalization for bleeding	1 (0.0%)	0 (0.0%)				
**1-year**	All-Cause Mortality	112 (3.4%)	71 (2.5%)	1.42 (1.05,1.92)	0.0234	1.76 (1.29,2.39)	0.0003
	All-Cause Hospital Readmission	831 (25.6%)	738 (25.6%)	1.00 (0.89,1.12)	0.9722	1.12 (0.99,1.26)	0.0653
	Hospitalization for heart failure	29 (0.9%)	29 (1.0%)	0.89 (0.53,1.49)	0.6540	0.80 (0.47,1.35)	0.4033
	Hospitalization for infection	133 (4.1%)	126 (4.4%)	0.94 (0.73,1.20)	0.6040	0.91 (0.71,1.17)	0.4684
	Hospitalization for stroke	18 (0.6%)	10 (0.3%)	1.60 (0.74,3.48)	0.2317	1.52 (0.70,3.30)	0.2932
	Hospitalization for ACS	0 (0.0%)	0 (0.0%)				
	Hospitalization for bleeding	1 (0.0%)	0 (0.0%)				

aOR – adjusted odds ratio

Intrathoracic

	Outcome (Pelvic Surgery)	Event rate (%)		Univariate		Multivariate	
		**Male**	**Female**	**OR (95% CI)**	**p-value**	**OR (95% CI)**	**p-value**
**30-day**	All-Cause Mortality	45 (0.3%)	19 (0.0%)	8.4 (4.91,14.36)	<.0001	3.68 (2.03,6.67)	<.0001
	All-Cause Hospital Readmission	1158 (6.5%)	2467 (3.9%)	1.71 (1.59,1.83)	<.0001	1.19 (1.09,1.31)	0.0003
	Hospitalization for heart failure	40 (0.2%)	37 (0.1%)	3.83 (2.45,5.99)	<.0001	0.93 (0.57,1.52)	0.7809
	Hospitalization for infection	176 (1.0%)	363 (0.6%)	1.72 (1.44,2.06)	<.0001	0.90 (0.72,1.13)	0.3584
	Hospitalization for stroke	16 (0.1%)	12 (0.0%)	4.72 (2.23,9.98)	<.0001	1.05 (0.50,2.23)	0.8955
	Hospitalization for ACS	0 (0.0%)	0 (0.0%)				
	Hospitalization for bleeding	2 (0.0%)	4 (0.0%)	1.77 (0.32,9.63)	0.5115	1.77 (0.32,9.63)	0.5115
**6-months**	All-Cause Mortality	197 (1.1%)	133 (0.2%)	5.29 (4.24,6.59)	<.0001	1.34 (1.04,1.72)	0.0216
	All-Cause Hospital Readmission	3242 (18.2%)	5156 (8.2%)	2.50 (2.38,2.62)	<.0001	1.36 (1.28,1.45)	<.0001
	Hospitalization for heart failure	113 (0.6%)	80 (0.1%)	5.02 (3.77,6.69)	<.0001	1.25 (0.91,1.71)	0.1724
	Hospitalization for infection	500 (2.8%)	674 (1.1%)	2.67 (2.38,3.00)	<.0001	1.12 (0.97,1.30)	0.123
	Hospitalization for stroke	52 (0.3%)	38 (0.1%)	4.85 (3.19,7.37)	<.0001	1.54 (0.98,2.40)	0.0592
	Hospitalization for ACS	2 (0.0%)	5 (0.0%)	1.41 (0.27,7.29)	0.6787	1.41 (0.27,7.29)	0.6787
	Hospitalization for bleeding	5 (0.0%)	6 (0.0%)	2.95 (0.90,9.66)	0.0742	2.95 (0.90,9.66)	0.0742
**1-year**	All-Cause Mortality	344 (1.9%)	256 (0.4%)	4.83 (4.10,5.68)	<.0001	1.23 (1.02,1.49)	0.0266
	All-Cause Hospital Readmission	4338 (24.3%)	7303 (11.6%)	2.46 (2.35,2.56)	<.0001	1.30 (1.23,1.38)	<.0001
	Hospitalization for heart failure	197 (1.1%)	132 (0.2%)	5.33 (4.27,6.65)	<.0001	1.38 (1.08,1.77)	0.0095
	Hospitalization for infection	737 (4.1%)	869 (1.4%)	3.09 (2.79,3.41)	<.0001	1.29 (1.14,1.46)	<.0001
	Hospitalization for stroke	72 (0.4%)	65 (0.1%)	3.93 (2.81,5.50)	<.0001	1.16 (0.81,1.65)	0.4171
	Hospitalization for ACS	6 (0.0%)	7 (0.0%)	3.03 (1.02,9.02)	0.0462	1.67 (0.51,5.42)	0.3931
	Hospitalization for bleeding	6 (0.0%)	8 (0.0%)	2.65 (0.92,7.65)	0.0708	2.65 (0.92,7.65)	0.0708

aOR – adjusted odds ratio

Pelvic

	Outcome (Orthopedic Surgery)	Event rate (%)		Univariate		Multivariate	
		**Male**	**Female**	**OR (95% CI)**	**p-value**	**OR (95% CI)**	**p-value**
**30-day**	All-Cause Mortality	96 (0.1%)	134 (0.2%)	0.82 (0.63,1.06)	0.1292	1.08 (0.82,1.42)	0.5702
	All-Cause Hospital Readmission	3122 (4.8%)	3262 (4.4%)	1.10 (1.04,1.15)	0.0004	1.18 (1.12,1.24)	<.0001
	Hospitalization for heart failure	173 (0.3%)	224 (0.3%)	0.88 (0.72,1.07)	0.2069	1.08 (0.88,1.34)	0.4467
	Hospitalization for infection	590 (0.9%)	674 (0.9%)	1.00 (0.89,1.11)	0.9667	1.09 (0.97,1.22)	0.1560
	Hospitalization for stroke	34 (0.1%)	63 (0.1%)	0.61 (0.41,0.93)	0.0223	0.80 (0.52,1.22)	0.2951
	Hospitalization for ACS	4 (0.0%)	2 (0.0%)	2.28 (0.42,12.43)	0.3418	2.28 (0.42,12.43)	0.3418
	Hospitalization for bleeding	1 (0.0%)	4 (0.0%)	0.29 (0.03,2.55)	0.2635	0.29 (0.03,2.55)	0.2635
**6-months**	All-Cause Mortality	356 (0.5%)	484 (0.7%)	0.84 (0.73,0.96)	0.0113	1.04 (0.90,1.20)	0.6232
	All-Cause Hospital Readmission	8845 (13.7%)	10092 (13.7%)	1.00 (0.97,1.03)	0.9338	1.08 (1.05,1.11)	<.0001
	Hospitalization for heart failure	528 (0.8%)	739 (1.0%)	0.81 (0.73,0.91)	0.0003	1.02 (0.90,1.15)	0.8005
	Hospitalization for infection	1484 (2.3%)	1728 (2.3%)	0.98 (0.91,1.05)	0.5396	1.03 (0.96,1.11)	0.3959
	Hospitalization for stroke	127 (0.2%)	198 (0.3%)	0.73 (0.58,0.91)	0.0058	1.02 (0.81,1.28)	0.8758
	Hospitalization for ACS	15 (0.0%)	11 (0.0%)	1.55 (0.71,3.38)	0.2666	1.49 (0.68,3.25)	0.3150
	Hospitalization for bleeding	5 (0.0%)	11 (0.0%)	0.52 (0.18,1.49)	0.2230	0.52 (0.18,1.49)	0.2230
**1-year**	All-Cause Mortality	542 (0.8%)	709 (1.0%)	0.87 (0.78,0.97)	0.0152	1.06 (0.94,1.20)	0.3329
	All-Cause Hospital Readmission	13891 (21.4%)	16691 (22.6%)	0.93 (0.91,0.96)	<.0001	1.02 (0.99,1.05)	0.1163
	Hospitalization for heart failure	776 (1.2%)	1108 (1.5%)	0.80 (0.73,0.87)	<.0001	0.99 (0.90,1.09)	0.8463
	Hospitalization for infection	2062 (3.2%)	2498 (3.4%)	0.94 (0.88,1.00)	0.0369	1.01 (0.95,1.07)	0.8394
	Hospitalization for stroke	231 (0.4%)	313 (0.4%)	0.84 (0.71,1.00)	0.0457	1.15 (0.97,1.37)	0.1073
	Hospitalization for ACS	32 (0.0%)	25 (0.0%)	1.46 (0.86,2.46)	0.1571	1.35 (0.80,2.28)	0.2631
	Hospitalization for bleeding	11 (0.0%)	13 (0.0%)	0.96 (0.43,2.15)	0.9294	0.96 (0.43,2.15)	0.9294

aOR – adjusted odds ratio

Orthopedic

	Outcome (Minor Surgery)	Event rate (%)		Univariate		Multivariate	
		**Male**	**Female**	**OR (95% CI)**	**p-value**	**aOR (95% CI)**	**p-value**
**30-day**	All-Cause Mortality	771 (0.8%)	513 (0.6%)	1.36 (1.22,1.52)	<.0001	1.29 (1.15,1.45)	<.0001
	All-Cause Hospital Readmission	10351 (10.7%)	7902 (9.0%)	1.20 (1.17,1.24)	<.0001	1.19 (1.15,1.22)	<.0001
	Hospitalization for heart failure	824 (0.8%)	588 (0.7%)	1.27 (1.14,1.41)	<.0001	0.98 (0.87,1.09)	0.6887
	Hospitalization for infection	2129 (2.2%)	1648 (1.9%)	1.17 (1.10,1.25)	<.0001	1.08 (1.01,1.16)	0.0171
	Hospitalization for stroke	144 (0.1%)	115 (0.1%)	1.13 (0.88,1.44)	0.3281	1.04 (0.81,1.33)	0.7740
	Hospitalization for ACS	9 (0.0%)	6 (0.0%)	1.35 (0.48,3.80)	0.5668	1.23 (0.44,3.45)	0.6988
	Hospitalization for bleeding	9 (0.0%)	8 (0.0%)	1.02 (0.39,2.63)	0.9752	0.85 (0.33,2.22)	0.7427
**6-months**	All-Cause Mortality	2370 (2.4%)	1643 (1.9%)	1.31 (1.23,1.40)	<.0001	1.31 (1.23,1.41)	<.0001
	All-Cause Hospital Readmission	23318 (24.0%)	18560 (21.2%)	1.18 (1.15,1.20)	<.0001	1.16 (1.14,1.19)	<.0001
	Hospitalization for heart failure	2159 (2.2%)	1679 (1.9%)	1.16 (1.09,1.24)	<.0001	0.90 (0.83,0.96)	0.0020
	Hospitalization for infection	5602 (5.8%)	4463 (5.1%)	1.14 (1.10,1.19)	<.0001	1.06 (1.02,1.11)	0.0031
	Hospitalization for stroke	403 (0.4%)	319 (0.4%)	1.14 (0.98,1.32)	0.0797	1.02 (0.87,1.18)	0.8332
	Hospitalization for ACS	31 (0.0%)	20 (0.0%)	1.40 (0.80,2.45)	0.2424	1.07 (0.61,1.89)	0.8071
	Hospitalization for bleeding	24 (0.0%)	15 (0.0%)	1.44 (0.76,2.75)	0.2643	1.44 (0.76,2.75)	0.2643
**1-year**	All-Cause Mortality	3389 (3.5%)	2371 (2.7%)	1.30 (1.23,1.37)	<.0001	1.32 (1.24,1.39)	<.0001
	All-Cause Hospital Readmission	29687 (30.6%)	24197 (27.6%)	1.15 (1.13,1.18)	<.0001	1.13 (1.11,1.15)	<.0001
	Hospitalization for heart failure	2985 (3.1%)	2315 (2.6%)	1.17 (1.11,1.23)	<.0001	0.89 (0.84,0.95)	0.0003
	Hospitalization for infection	7414 (7.6%)	5946 (6.8%)	1.14 (1.10,1.18)	<.0001	1.05 (1.01,1.09)	0.0110
	Hospitalization for stroke	636 (0.7%)	486 (0.6%)	1.18 (1.05,1.33)	0.0056	1.04 (0.92,1.17)	0.5685
	Hospitalization for ACS	49 (0.1%)	41 (0.0%)	1.08 (0.71,1.63)	0.7209	0.82 (0.54,1.25)	0.3583
	Hospitalization for bleeding	37 (0.0%)	24 (0.0%)	1.39 (0.83,2.33)	0.2078	1.39 (0.83,2.33)	0.2078

aOR – adjusted odds ratio

Minor
