# Supplementary material for: Sex differences in post-operative outcomes following non-cardiac surgery
Source: PLoS One. 2023 Nov 1;18(11):e0293638. doi: 10.1371/journal.pone.0293638 (PMC10619824; doi:10.1371/journal.pone.0293638)
Supplement: S1 Fig — (PDF) [file pone.0293638.s005.pdf]

Supplemental Figures

Supplemental Figure 1: Causes of Re-Hospitalization at 30-days, 6-month, and 1-year based on ICD-10 codes.

A.

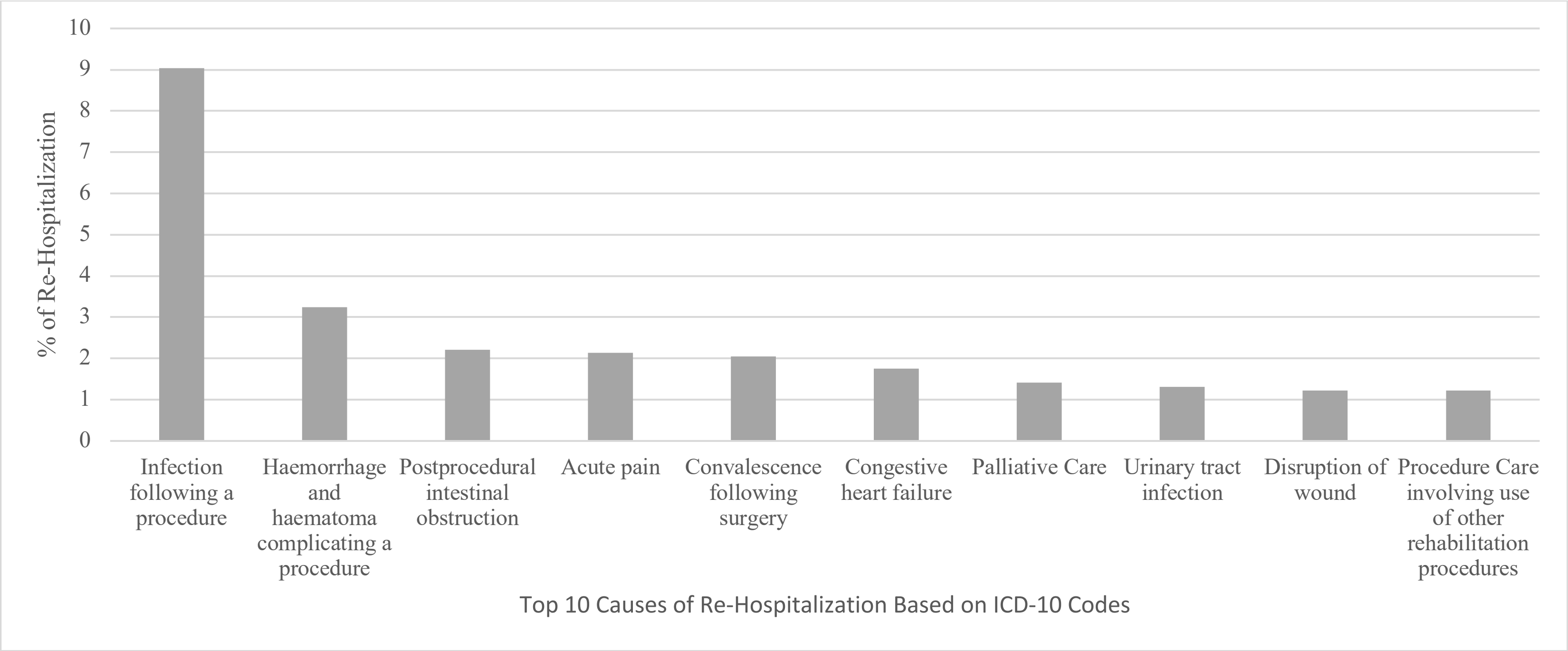

10
9
8
7
6
5
4
3
2
1
0
% of Re-Hospitalization
Infection following a procedure
Haemorrhage and haematoma complicating a procedure
Postprocedural intestinal obstruction
Acute pain
Convalescence following surgery
Congestive heart failure
Palliative Care
Urinary tract infection
Disruption of wound
Procedure Care involving use of other rehabilitation procedures
Top 10 Causes of Re-Hospitalization Based on ICD-10 Codes

B.

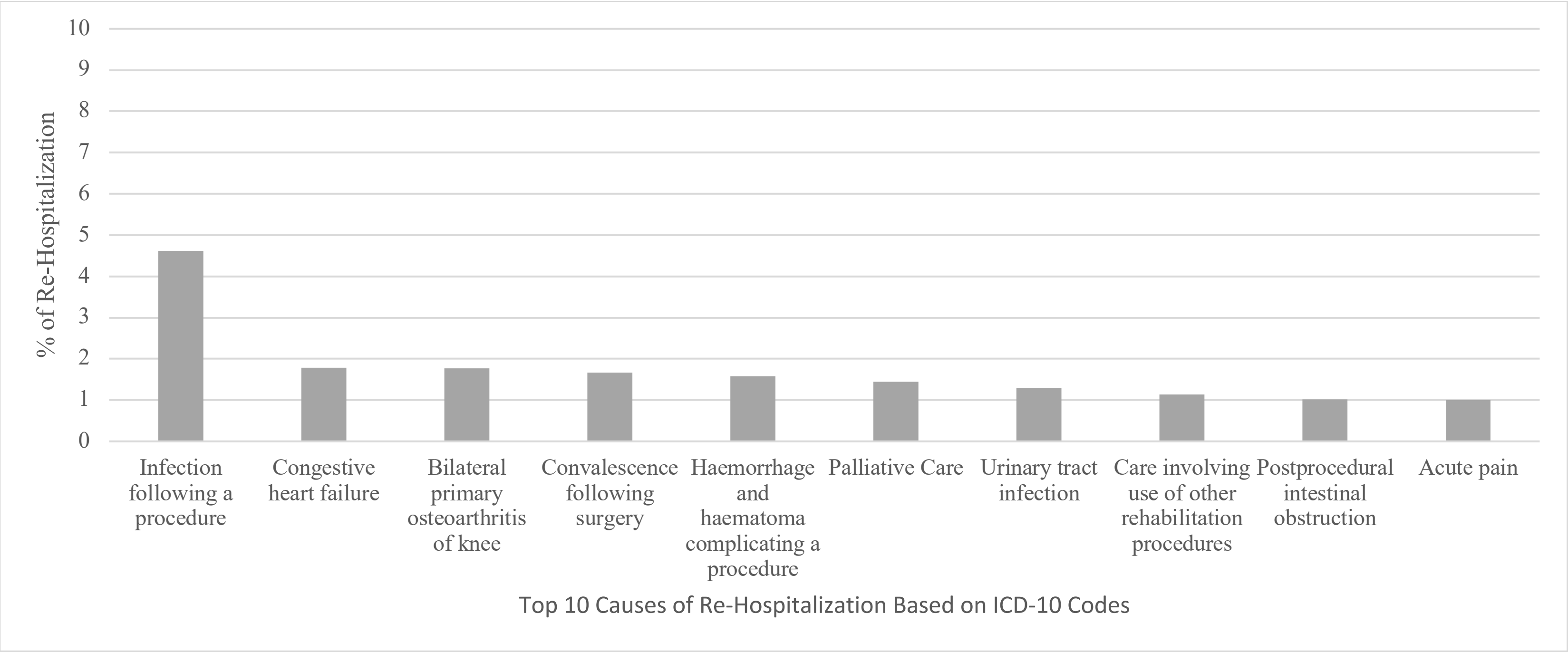
% of Re-Hospitalization
0
1
2
3
4
5
6
7
8
9
10
Infection following a procedure
Congestive heart failure
Bilateral primary osteoarthritis of knee
Convalescence following surgery
Haemorrhage and haematoma complicating a procedure
Palliative Care
Urinary tract infection
Care involving use of other rehabilitation procedures
Postprocedural intestinal obstruction
Acute pain
Top 10 Causes of Re-Hospitalization Based on ICD-10 Codes

C.

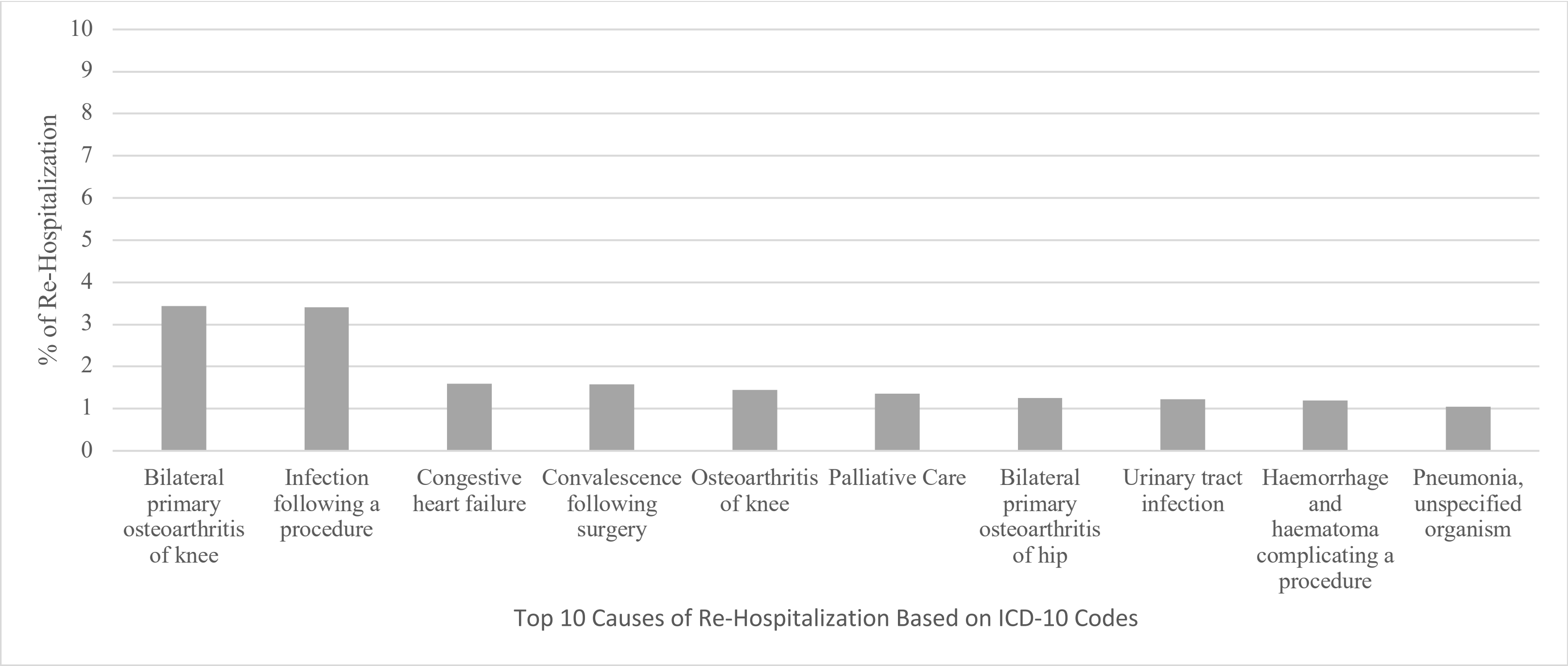
% of Re-Hospitalization
0
1
2
3
4
5
6
7
8
9
10
Bilateral primary osteoarthritis of knee
Infection following a procedure
Congestive heart failure
Convalescence following surgery
Osteoarthritis of knee
Palliative Care
Bilateral primary osteoarthritis of hip
Urinary tract infection
Haemorrhage and haematoma complicating a procedure
Pneumonia, unspecified organism
Top 10 Causes of Re-Hospitalization Based on ICD-10 Codes
